# Evidence on the reach and impact of the social physical activity phenomenon *parkrun*: A scoping review

**DOI:** 10.1016/j.pmedr.2020.101231

**Published:** 2020-11-05

**Authors:** Anne Carolyn Grunseit, Justin Richards, Lindsey Reece, Adrian Bauman, Dafna Merom

**Affiliations:** a Prevention Research Collaboration, Sydney School of Public Health, University of Sydney, Camperdown, Australia; b Faculty of Health, Victoria University Wellington, Wellington, New Zealand; c Physical Activity and Health, School of Science and Health, Western Sydney University, Parramatta, Australia

**Keywords:** PRISMA-Sc(R), Preferred Reporting Items for Systematic Reviews and Meta-Analyses Extension for Scoping Reviews, RE-AIM, Reach, efficacy, adoption, Implementation, maintenance, Physical activity, parkrun, Review, Health promotion, Scaling up, Qualitative, RE-AIM

## Abstract

The aim of this study was to conduct a scoping review of parkrun literature for evidence of its reach, health impact and appeal whilst identifying gaps for future research. We used the Preferred Reporting Items for Systematic Reviews and Meta-Analyses Extension for Scoping Reviews (PRISMA-ScR). Six databases were searched according to search terms set a priori. Empirical studies of any design were included if they provided data on participation in, or benefits of, parkrun and were in English. Two authors conducted the searches independently and screened results by title and abstract, followed by full text reviews. A total of 235 records were screened and 15 studies were eligible; 12 were conducted in the UK and three in Australia. Seven were qualitative interview studies, six were quantitative, and two used mixed methods. parkrun reaches groups traditionally underrepresented in other organised sports or physical activity such as women, the insufficiently active and those aged over 35 years. Participants showed sustained improvements in fitness, physical activity levels, and body mass index with a dose–response effect with participation frequency. Qualitative data shows parkrun’s location in pleasant environments with opportunities for informal social interaction engages priority groups such as individuals with mental health issues, women and children. The small evidence base suggests parkrun has good reach, and can positively impact participants’ health and wellbeing. The data, however, are currently UK-centric and gaps in research on non-participants, long term health impacts and operationally relevant factors should be addressed.

## Highlights

• parkrun research shows health value and a model which suits participants.
• parkrun engages traditionally under-represented populations in physical activity.
• parkrun participation has a dose–response relationship with improved fitness.
• Future research should examine discontinuing participants and non-participants.

## 1 Introduction

The health benefits of physical activity are well established (Sallis et al., 2016). However, there is limited published evidence for interventions that are effective in enhancing population physical activity levels and are demonstrably scalable and sustainable. Despite an abundance of researcher-led examples of small-scale effective physical activity interventions, most of these have failed to be sustained at scale in pragmatic real-world settings (Reis et al., 2016). Practice-based research, where effective interventions already operating at scale are systematically examined for the factors that make them successful, is an innovative approach to moving the field forward and ensuring research findings are translatable (Ding et al., 2019).

Since 2004, parkrun has organised free, weekly timed 5 km run/walks that started as a single event in Bushy Park, England and has now expanded across 22 countries every Saturday (www.parkrun.com). As a volunteer-run community-based event, parkrun prides itself on an ethos of inclusivity and has succeeded in spreading its reach by increasing participant numbers at existing events and replicating the same model in places as diverse as South Africa, Iceland and Japan (Reece et al., 2019). parkrun growth appears to have largely been organic across countries supported by the parkrun administration, and yet stands out among other physical activity interventions in terms of its scalability, sustainability, accessibility and potential to disrupt the socio-economic gradient of health behaviours (Wiltshire and Stevinson, 2018). In two reviews of scaled-up public health physical activity interventions, no program had operated internationally through a central administration nor achieved dissemination solely through civil society (Reis et al., 2016, Schell et al., 2013); more than 4 million people have participated parkrun worldwide in over 1800 locations (www.parkrun.com). However, the emerging peer-reviewed evidence base examining the impact and underlying mechanisms of parkrun remains uncollated and unexamined as an example of successful “real world” sustained social physical activity interventions at scale.

The aim of this scoping review is to systematically map the current evidence for parkrun in terms of its: 1) Reach and diversity of participation; 2) Impact and implications for participants’ health and wellbeing; 3) Mechanisms for parkrun’s appeal and dissemination across populations. A scoping rather than systematic review is appropriate as this is an emerging field comprising a small number (n = 15) of published empirical studies that are heterogeneous in terms of methods, measures and foci (Munn et al., 2018). Moreover, our purpose is to map what is currently known about parkrun and identify gaps in knowledge/evidence to guide researchers of parkrun and mass participation physical activity interventions, aims better suited to a scoping review (Munn et al., 2018, Arksey and O'Malley, 2005).

## 2 Methods

### 2.1 Approach

We conducted a scoping review of parkrun evidence to assess the nature and extent of the existing literature (Grant and Booth, 2009), summarise and disseminate findings and identify gaps to guide future research (Arksey and O'Malley, 2005). We followed established guidelines for scoping reviews (Tricco et al., 2018).

### 2.2 Eligibility criteria

We included peer reviewed studies of any design as long as empirical data on the participation in, or benefits of, parkrun was the focus of the study. We excluded conference abstracts that were not published as full articles.

### 2.3 Information sources and search terms

**Fig. 1:**
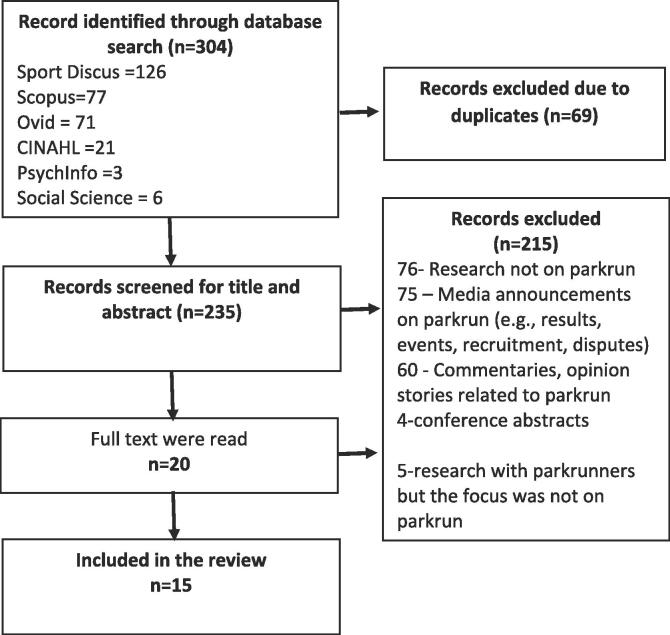
PRISMA chart flow of study selection.

The terms “parkrun” or “ParkRun” or “parkrun*” were used in a search of six databases: Sport Discus, Scopus, Ovid databases [EMBASE + MEDLINE], CINAHL, PsychInfo and Social Science database (See Fig. 1). We limited our search from the year 2004 (when parkrun was initiated) to December 2019. No other limits were imposed. Further manual searches were conducted of the parkrun research publications webpage (Advanced Wellbeing Research Centre, 2020b) and the reference lists of included papers.

### 2.4 Screening and study selection

Two authors (AG, DM) conducted the database search independently. After removing duplicates, two authors (AG, DM) then screened by title and abstract. The full text of all articles was then screened for all records classified as included by at least one of the screeners. Final decisions on inclusion were made by consensus.

### 2.5 Data Items and synthesis

We extracted data on article characteristics (author, year, country of origin), study characteristics (study design, topic focus, population, number of parkruns involved) and the main findings (Table 1). We summarised study findings under the three aims outlined above. A number of studies reported findings relevant to more than one aim and therefore may appear in multiple sections of the results.

**Table 1:** Characteristics of included studies.

Author	Year	Country	Data source	Topic focus	Population	n (people); n (parkruns)*	Main findings
(Stevinson and Hickson, 2014)	2014	UK	Quantitative cross-sectional survey + matched administrative data	Profile of participants, fitness outcomes and participation, subjective outcomes (physical & mental)	Adult parkrunners 18+ (NFS)	7308; 130	- Majority of sample were non-runners - Physical (fitness, weight and health) and psychological (mental wellbeing and confidence for running) benefits were reported significantly more frequently among participants who were not regular runners on joining parkrun
(Stevinson et al., 2015)	2015	UK	Qualitative in-depth interviews	Personal motives and experiences for initial and continued participation	Adult parkrunners NFS	48, NR	- Freedom and reciprocity were overarching themes - Accessibility, inclusivity and anticipated fitness, weight and health benefits prompt initial participation - Continued participation was attributed to feeling accepted, lack of pressure, developing social ties and confidence, and opportunities to give back (volunteering)
(Rogerson et al., 2016)	2016	UK	Quantitative immediate pre-post surveys + matched administrative data	Impact of green exercise on affective outcomes	parkrunners completing the course on collection days	331, 4	- Significant acute improvements in psychological wellbeing found post-run - Run enjoyment and performance in relation to expectation positively predicted improvement in self-esteem - Individuals’ nature-relatedness, being female and run enjoyment were positively associated with improvement in mood pre to post-run
(Grunseit et al., 2018)	2018	Australia	Quantitative cross-sectional survey	Personal wellbeing, benefits of parkrun and wellbeing	Adult parkrunners (NFS)	865, 96	- Higher satisfaction with health for male, >45 years and parkrunners overall vs general population equivalents - Positive association of *satisfaction with life as a whole* with perceived mental health benefits of parkrun for women - Positive association between perceived community connection benefits of parkrun for men and mental health benefit for women and global index of personal wellbeing
(Hindley, 2018)	2018	UK	Participant observation, in-depth interviews, on-line cross-sectional survey	parkrun as a social practice	Adult parkrun runners and volunteers (NFS)	19 interviewees, 235 survey participants, 1	- Participation in parkrun provides an inclusive leisure space for casual sociability, and facilitates a shared experience of exercising with others
(Wiltshire et al., 2018)	2018	UK	Qualitative in-depth interviews	Examine processes of how parkrun is understood as a health practice	Adult parkrunners with little or no running experience	19, 16	- parkrun allows participants to work on their personal body projects but as part of a group process which helps mitigate the individualising effects of the self-responsibility health discourse - parkrun allows previously inactive subjects to move from feeling excluded from sport and physical activity, to being included because parkrun opens up a diverse range of running practices (walk-running, jogging, competition)
(Wiltshire and Stevinson, 2018)	2018	UK	Qualitative in-depth interviews	Understand the role of social capital in parkrun	Adult parkrunners with little or no running experience	20, 17	- Social ties initiated parkrun participation - Participants invest and benefit from practical and emotional support within the wider parkrun community - parkrun mobilises the flow of cultural capital (advice and guidance on running and general health) to low SES groups
(Cleland et al., 2019)	2019	Australia	Quantitative cross-sectional survey	Participation, correlates of participation	Tasmanian participants, adults (NFS)	371, 3	- Being married/partnered and parkrun outside Tasmania associated with higher parkrun participation, inverse relationship with education level - Self-efficacy, enjoyment, intention to participate in parkrun over the next 2 weeks, perceived social benefits, family support for parkrun, and “knowing lots of people who do parkrun” was positively associated with parkrun participation
(Fullagar et al., 2019)	2019	UK	Qualitative and quantitative data from cross-sectional survey	Participation for non-traditional subpopulations/marginalised group	parkrun volunteers, organisers and participants	Survey 655, 4 In-depth interviews 19, 4	− 70% of surveyed participants thought parkrun is inclusive, but there were exception sites (e.g., London) where this was not the case - Three strategies to increase inclusiveness were revealed: 1) promoting the parkrun ‘ethos’ in the media with different emphasis 2) develop joined-up relationship with local government and non-government organisations to support better parkrun promotion 3) fostering an inclusive culture within parkrun
(Morris and Scott, 2019)	2019	UK	Qualitative in-depth interviews	Experience of parkrun for people with history of mental health issues	Adult parkrunners 28–65 with mental health difficulties (>10 parkruns)	20; NR	- parkrun was reported to be beneficial to psychological wellbeing through the sense of community, social opportunities and opportunities for accomplishment - parkrun helped improve mood, increase confidence and self-esteem and reduce isolation
(Quirk and Haake, 2019)	2019	UK	Qualitative in-depth interviews	Evaluation of the impact of parkrun long-term physical, intellectual or mental chronic disease ambassadors	Outreach Ambassadors in parkrun: running or volunteering for everyone - PROVE) project	13; NA	- Volunteer outreach ambassadors were felt to be effective in promoting inclusivity among people with long term chronic illnesses Value was felt to lie in challenging stereotypes, making parkrun sensitive to the needs for people with chronic illnesses
(Sharman et al., 2019)	2019	Australia	Qualitative in-depth interviews	Individual, social and environmental factors associated with participation	Adult Tasmanian parkrunners	10, NR	- Appeal of parkrun spans the social-ecological model through: reciprocal support, fitness, attendance rewards and incentives, social opportunities, sense of community, inclusivity, accessibility, feeling safe and off-road environment in which to be active. - parkrun name may imply fast running to some
(Stevens et al., 2019)	2019	UK	Longitudinal survey + matched administrative data	Group/social identification, group cohesion, exercise-specific satisfaction, life satisfaction	Adult parkrunners 18+ (NFS)	289; NR	- Significant positive relationship between parkrun group identification and: life satisfaction, subsequent parkrun participation, and satisfaction with parkrun experience - no relationship between parkrun participation and life satisfaction
(Stevinson and Hickson, 2019)	2019	UK	Prospective survey + matched administrative data	Change in physical activity, weight, happiness and stress over 12 months	New adult parkrun registrants	354, 1	- Increases in total PA and fitness at 6 and 12 months, more pronounced in those who were low active at baseline - Overweight and obese participants made the largest relative weight change - Happiness significantly increased and stress and depression decreased - Small but significant correlation between number of runs and lower BMI, stress and increases in happiness
(Stride et al., 2019)	2019	UK	Qualitative in-depth interviews	Experience of women as volunteers in sports/leisure	Female adult parkrun volunteers	NR, 11	- Volunteer roles at parkrun were taken up by women along traditional gender lines - the parkrun model allowed for some elevation of the value of the traditionally feminine (nurturing/support) roles - the variety of roles in parkrun were flexible to accommodate the caring responsibilities and changes in life circumstances experienced by women

* NR – not reported.

## 3 Results

Fig. 1 describes the PRISMA chart flow of study selection. A total of 304 records were combined from all databases and after removing 69 duplicates, 235 records were screened for title and abstract. Exclusions were primarily due to: research on other unrelated parks or runners (35%); parkrun announcements such as features in Athletes Weekly (34%); commentaries on parkrun with no primary research (28%), conference abstracts (1.5%), with the remaining papers involving parkrun samples, but no specific research on or about parkrun itself. Table 1 details the characteristics of included studies. Most were conducted in the UK (n = 12) and three concerned Australian samples.

### 3.1 Reach and participant profile

Four papers described the participant (who may run/walk and/or volunteer) profile of parkrunners (Stevinson and Hickson, 2014, Cleland et al., 2019, Fullagar et al., 2019, Grunseit et al., 2019). All studies recruited individuals directly through parkrun (rather than from the general population) and were cross-sectional analyses. Two studies were conducted in Australia, one nationally (Grunseit et al., 2018), the other in one state (Tasmania) (Cleland et al., 2019). Of two UK studies, one recruited widely across the nation (Stevinson and Hickson, 2014) and the other targeted just four sites (Fullagar et al., 2019).

#### 3.1.1 Demographic profile

The two Australian surveys were comprised of more females than males (58.4% (Cleland et al., 2019), 61.5% (Grunseit et al., 2018)), but the genders were almost equal in the UK based studies (Stevinson and Hickson, 2014, Fullagar et al., 2019). For all four, the majority of participants were aged between 35 and 54 years (Stevinson and Hickson, 2014, Cleland et al., 2019, Fullagar et al., 2019, Grunseit et al., 2019). One study compared the survey sample to the distribution of registered parkrunners at the sampled parkruns (n = 96) finding the distribution of registrants was younger than those surveyed (44% < 35 years registrants vs. 22% survey sample), but the proportion of female registrants was close to that of the survey sample (57.4% vs 61.5%) (Grunseit et al., 2018). A bias in parkrun engagement towards higher socioeconomic groups was observed when assessed by employment (Stevinson and Hickson, 2014, Cleland et al., 2019), education (Cleland et al., 2019), or income (Fullagar et al., 2019) Almost all parkrunners sampled were white (Stevinson and Hickson, 2014), and English speakers at home (Cleland et al., 2019).

#### 3.1.2 Physical health and wellbeing

Two studies reported participants weight status and showed that whilst the majority were in the healthy weight range (>18 BMI ≤ 25), the event also attracts a significant proportion of overweight or obese individuals (43.5% (Cleland et al., 2019); 33.2% (Stevinson and Hickson, 2014)). The rates for both studies were highest among non-regular runners non-walker/runners (Stevinson and Hickson, 2014, Cleland et al., 2019). Self-rated health was excellent or very good for 57% and only a small proportion (4%–7%) reported an injury, illness or disability (Stevinson and Hickson, 2014, Cleland et al., 2019). Grunseit et al. (2018) compared Australian parkrunners with national population norms for perceived physical health and personal wellbeing and found higher ratings for parkrunners than general population, particularly amongst older (>45 years) and male parkrunners. Younger (18–24 years) and male participants also ranked below age equivalent population norms on other domains such as current achievements, personal relationships and life as a whole (Grunseit et al., 2018).

#### 3.1.3 Physical activity levels and parkrun participation

Physical activity levels of parkrunners were relatively high compared with population norms, with 57% achieving health enhancing levels in the UK study (Stevinson and Hickson, 2014) and a median of 6 h of total physical activity a week in the Australian study (Cleland et al., 2019). In the UK sample, parkrun attracted a significant proportion (25%) of previous non-runners (Stevinson and Hickson, 2014). The Australian study estimated non-walkers and/or non-runners at 15% upon parkrun registration (Cleland et al., 2019). Previous non-runners or walkers were more likely to be female and report higher body weight and lower overall physical activity (Stevinson and Hickson, 2014, Cleland et al., 2019).

Two studies reported regularity of parkrun participation in their survey samples. Fullagar et al. (2019) found that at least 80% of their sample participated at least monthly (43%) or weekly (37%) across the four sampled sites (Fullagar et al., 2019). Stevinson’s wider sample included at least half who had already attended at least 51 weeks (IQR:21–101) but importantly, median attendance regularity (defined as a percentage of the number of Saturdays attended since their first run) was higher among initial non-runners (50.0%) than occasional (42.9%) and regular (37.5%) runners (Stevinson and Hickson, 2014). Correlates of participation were investigated in the Australian study and being married was associated with higher participation and higher education associated with lower participation (Cleland et al., 2019).

#### 3.1.4 Interventions/strategies to increase parkrun inclusivity

Two studies from the UK evaluated interventions and strategies aimed at increasing inclusivity for parkrun (Fullagar et al., 2019, Quirk and Haake, 2019). The qualitative study by Quirk and Haake (2019) evaluated a parkrun participation strategy, PROVE (**p**arkrun: **r**unning **o**r **v**olunteering **f**or **e**veryone) which used volunteer Outreach Ambassadors to increase parkrun participation among people living with a long term chronic illness (e.g., dementia, heart conditions, asthma, mental health) (Table 1). Respondents felt PROVE gave structure and a vehicle for parkrun inclusion among this population. PROVE ambassadors noted that having suitable personnel and external engagement were key to promoting inclusivity within the constraints of what could be expected from a volunteer workforce. Another study of perceptions on and strategies for increasing inclusivity of non-traditional participants/marginalized groups found respondents commonly felt parkrun was inclusive, but in some locations (e.g., London) diversity in terms of ethnicity and religion was lacking. The authors also noted the tension between greater inclusivity and volunteer capacity (Fullagar et al., 2019).

### 3.2 Impact of parkrun

#### 3.2.1 Physical effects

The impacts of parkrun on physical activity outcomes were assessed by two UK quantitative studies: one cross-sectional (Stevinson and Hickson, 2014), and one prospective longitudinal design (Stevinson and Hickson, 2019), both of which matched survey responses with (longitudinal) parkrun administrative data. Stevinson and Hickson (2014) surveyed a large representative sample of parkrunners (n = 7308 adults) and found improvements in cardiorespiratory fitness from first participation occasion to their fastest parkrun time in the survey year; age-adjusted time improved on average by 10%. Greatest improvement (15.8%) was among non-runners at registration, followed by ‘occasional runners’ (11.3%) and least by ‘regular runners’ (7.6%), likely a ceiling effect. parkrun performance improvement was moderately to strongly correlated (r = 0.55) with regularity of participation. Survey participants perceived parkrun contributed to improved fitness.

Arguably, the strongest evidence for the physical benefits of parkrun was captured by Stevinson and Hickson (2019) in their prospective study of n = 878 newly registered adults (Stevinson and Hickson, 2019). At 6 months, 63% and at 12 months 53% were still attending parkrun with no statistically significant differences at baseline between survey completers and non-completers (Stevinson and Hickson, 2019). New registrants were already highly active with only 8.8% (n = 31) classified below the minimum recommendation for moderate-to-vigorous–intensity physical activity (MVPA). Yet vigorous-intensity and total MVPA increased significantly (by 20.8 min and 76.9 min, respectively) at 6 months. At 12 months total MVPA remained higher than baseline whereas vigorous-intensity activity returned to baseline levels. Importantly, among those who started below recommended levels, mean total MVPA (194 min) and vigorous-intensity PA (60 min) per week were higher than baseline. An Australian qualitative study corroborated these findings reporting that previously low active participants felt that parkrun facilitated expanding or re-entry to physical activity participation (Sharman et al., 2019). Stevinson and Hickson (2019) also found that anthropometric measures similarly improved with a significant reduction in BMI at 12 months (-0.3 (-0.2, −0.5)) compared with baseline, greatest among those who were overweight (−0.7, (−0.4, −1.0)) (Stevinson and Hickson, 2019).

#### 3.2.2 Mental health and wellbeing

The impacts of parkrun on mental wellbeing were assessed by one qualitative study (Morris and Scott, 2019) and three quantitative studies: one cross-sectional design (Stevinson and Hickson, 2014); one longitudinal design (Stevinson and Hickson, 2019); and one pre-post study examined the acute affective response to parkrun participation (Rogerson et al., 2016). All were conducted in the UK.

Morris and Scott (2019) qualitatively investigated the perceived impact of parkrun on people who were currently or had previously experienced mental health difficulties and had participated as a walker/runner or volunteer in at least 10 parkruns (Morris and Scott, 2019). People associated parkrun with enhanced feeling of wellbeing through reducing social isolation, depression, anxiety, stress and increasing confidence. Three key affordances of parkrun were proposed to underpin these outcomes: “sense of achievement”, “it’s for everyone”, and “connecting with others”. Quantitative analyses showed the greatest gains were reported by those who were not regular runners when they started parkrun and also among those who participated in parkrun more often (Stevinson and Hickson, 2014). A longitudinal study of ~350 people found significant improvements in happiness, stress levels and the number of participants classified as at-risk for depression over a 12 month period (Stevinson and Hickson, 2019). As for the earlier study, changes were larger among those who participated in parkrun more regularly and reductions in stress were greater for novice runners (Stevinson and Hickson, 2019).

Rogerson et al. (2016) found that participating in parkrun had immediate positive impacts on self-esteem, stress and mood. Perceived enjoyment, and satisfaction with performance were key predictors of the change in self-esteem; changes in mood were associated with gender, perceived enjoyment and how connected the person felt to nature (Rogerson et al., 2016). However, these factors only accounted for ~ 10% of the changes observed in self-esteem and mood.

### 3.3 Mechanisms for parkrun participation and impacts

Six studies primarily focussed on the mechanisms for initial and continuing parkrun attendance (Wiltshire and Stevinson, 2018, Stevinson et al., 2015, Hindley, 2018, Wiltshire et al., 2018, Sharman et al., 2019, Stevens et al., 2019). One further paper explored gender in volunteering at parkrun, through comparisons across three different contexts (parkrun, cycling, leisure organisations (Stride et al., 2019). Except for one study qualitative methods were used (Stevens et al., 2019); Hindley (2018) used participant observation and online surveys in addition to qualitative interviews (Hindley, 2018). Two studies adopted a psychological or social-psychological frame, taking parkrunners’ reports of their reasons for their participation largely at face value and organising the findings thematically (Stevinson et al., 2015, Sharman et al., 2019). The remainder (Wiltshire and Stevinson, 2018, Wiltshire et al., 2018, Stride et al., 2019) took more critical approaches.

Two qualitative interview studies examining reasons for participation in parkrun had consistent findings despite being conducted in different countries (Stevinson et al., 2015, Sharman et al., 2019). Both concluded that the free, low-demand participation and diversity in parkrun participants’ ability contributed to initial participation; a sense of achievement and the social nature of the events, development of social ties, sense of community and opportunities for volunteering (reciprocity) led to continued attendance. Stevens et al. (2019) tested the effect of group identification quantitatively and found positive relationships with satisfaction with parkrun experience, subsequent participation and life satisfaction.

Resonating with Stevens’ quantitative analysis, Hindley (2018) argued that parkrun facilitates and confirms a recreational running identity through parkrun-specific norms, behaviours and language (Hindley, 2018). For example, the milestone t-shirts signify achievements in attendance and membership of a collective recognisable to other network members. Uniquely, Hindley (2018) critiques whether parkrun may be viewed as a “third place” for social interaction (third to home and work); its attractions have similarities to other “third place” settings such as incidental interactions, mutual support and definition by regulars, but also differences such as being temporary, generative of long-term commitment, and participants’ desire to promote parkrun in the community. He concludes that parkrun is a meaningful leisure phenomenon beyond a physical health promoting activity.

Two studies targeted adult parkrunners with little or no previous running experience to examine whether parkrun could disrupt social inequalities in physical activity promotion (Wiltshire and Stevinson, 2018, Wiltshire et al., 2018). According to Wiltshire and Stevinson (2018) social capital is both drawn upon by parkrun (social ties facilitate initiation, volunteers run the events) and generated by it through the social networking opportunities. Cultural capital consequently flows from high to low cultural capital groups through the exchange of knowledge about running, health and other physical activities. parkrun may therefore mobilise resources otherwise unavailable to those with low social capital, although these groups must first clear the hurdle of initiation (Wiltshire and Stevinson, 2018).

A second paper also addresses the socially redistributive potential of parkrun but this time as a health practice (Wiltshire et al., 2018). The authors contend that the low-competitive, social and inclusive nature of the parkrun ethos serves to position people’s “personal body projects” as a collective rather than individual responsibility. Similarly, Stride et al. (2019) argue that parkrun also disrupts traditional gender practices through volunteering. For example, tasks such as tail runner, seen as a comfortable role for women, are elevated in importance in the parkrun model (Stride et al., 2019). Further, the low commitment, time–limited, variety of volunteer roles and potential for children to also attend parkrun make volunteering available to women where their family responsibilities may preclude it in other sports/leisure contexts.

## 4 Discussion

The reach and potential impact of parkrun has been noted anecdotally for some time, but the empirical evidence remains limited. Over four million people have participated in parkrun across five continents, and expansion continues (www.parkrun.com/countries). Our review demonstrates a UK-centric evidence base that shows health value and a model that works well for those who have been accessed by the research conducted thus far. According to the studies reviewed, mechanisms including the timed component, informal social nature, location in green space, and the physical activity itself, collectively offer participants ways of testing and developing their fitness, making social connections, feeling valued, and disrupting hegemonic discourses around gender and physical competence. Similar to Reis et al. (2016), below we use the RE-AIM framework to integrate across the findings of the studies reviewed for important pointers for future parkrun research and the field of population level physical activity interventions more broadly.

### 4.1 Reach

parkrun is one of the few global physical activity initiatives that is suitable for individuals across the lifecourse, and of different levels of physical and mental health. For example, research illustrates parkrun's ability to engage older people and women and girls, who traditionally are under-represented in organised sport and physical activity (Guthold et al., 2018, Hanlon et al., 2019). The average parkrun finishing time is increasing, with greater participation of non-runners who walk the course (parkrun UK, 2017). However, as yet no study examined the correlates of parkrun walkers and whether they are different to other participants, despite reaching marginalised groups that are typically less active (e.g. women, ethnic minorities, low income, older people, those with disabilities or illness) (Quirk and Haake, 2019).

### 4.2 Effectiveness

There appears to be a dose–response relationship for regular parkrun participation and improved fitness that is most pronounced in those previously insufficiently active (parkrun UK, 2017, Ecclestone et al., 1998). Both quantitative and qualitative data indicate that improving fitness and anthropometric outcomes are key motivating factors for commencing and continuing participation in parkrun. However, the dearth of longitudinal evidence beyond 12 months and objective health measures hampers drawing definitive conclusions. Social outcomes also contribute to maintain participation (Stevinson and Hickson, 2014, Sharman et al., 2019). The improvement in mental wellbeing both in the short (Rogerson et al., 2016) and longer (Stevinson and Hickson, 2019) term is another positive finding of the current studies, but the research is yet to examine what mediates that improvement. A few authors noted potential wider economic effects at the community level (Sharman et al., 2019), redistribution of social capital (Wiltshire and Stevinson, 2018), and re-setting social norms and discourses on gender (Stride et al., 2019), health and exercise performance (Wiltshire et al., 2018).

### 4.3 Adoption

parkrun has been widely adopted (22 countries, ≈1800 events, wwwparkrun.com), but there currently is no data at the population level on participation in defined geographical or census areas. Further, there is no published research that critically examines the patterns and system level determinants of parkrun dissemination. Historically, parkrun’s growth has been largely organic, with much of the impetus for establishing new events grounded in community demand (Sperandei et al., 2016). Strategies linking parkrun dissemination to other institutions are now emerging (e.g. primary care through “parkrun practices” (Fleming, 2019), peak sports bodies (Orienteering Australia, 2019), health insurance (parkrun AU, 2019) and corrections centres (Reece et al., 2019). Fullagar et al. (2019) recommended partnering with non-government organisations to increase parkrun inclusiveness. Recently the Irish National Cancer Control was established to promote cancer prevention awareness within parkrun, with clear message: “Bring a friend to parkrun on the 3rd February for World Cancer Day” (Lyng et al., 2018). Whilst parkrun adoption and linkages with other health initiatives and institutions is clearly occurring, we are unaware of any formal evaluations of these partnerships.

### 4.4 Implementation

Implementation currently receives little systematic attention in the parkrun peer review literature. parkrun *prima facie* operates a very consistent model worldwide; events must meet a number of requirements (e.g., 5 km distance, run by volunteers, events are timed, each event has a pro forma description on the parkrun website) which do not change regardless of location. An analysis of event establishment processes and ongoing management may reveal a plethora of strategies by which parkruns sustain themselves and deliver a locally relevant event. One narrative emerging from the qualitative studies demonstrates that voluntary labour forms an integral part of the reciprocity that underpins the sustainability of parkrun (Stevinson et al., 2015, Sharman et al., 2019), but only two studies purposefully sampled volunteers (Fullagar et al., 2019, Stride et al., 2019).

### 4.5 Maintenance

We found some evidence that parkrun effectively retains participants and contributes to the maintenance of health (Stevinson and Hickson, 2019). The parkrun model aligns with evidence demonstrating the value of rewarding participation rather than performance for maintaining both walking/running and volunteering (Stevinson et al., 2015, Sharman et al., 2019). The retention rate of 63% of parkrunners after 12 months (Stevinson and Hickson, 2014) compares well with estimates for unsupervised gym membership where 63% of new members ceased activities before the third month and only 3.7% were retained for more than 12 months (Sperandei et al., 2016). Similarly, an observational study of seniors adherence to community-centre PA classes, found 51% remained after the first year of enrolment (Ecclestone et al., 1998). The sustainability of the short term impact of parkrun on mental wellbeing outcomes remains unknown (i.e. how long do stress levels remain lower and self-reported mood and self-esteem remain elevated). The subsequent impact of sustained participation on numerous health outcomes can be extrapolated from the broader physical activity and health literature, but has not been specifically assessed for parkrun.

### 4.6 Strengths & limitations

Our scoping review of the research on parkrun is the first to be undertaken and has employed an evidence based framework to draw out practice-based evidence from parkrun relevant for scaling up physical activity interventions (Reis et al., 2016). We used an established approach to conducting the search and synthesising data appropriate to a scoping review (Arksey and O'Malley, 2005, Tricco et al., 2018). Although it is likely that we captured all empirical studies, we did not include numerous commentaries, blogs and non-peer review articles. Further, we restricted our included studies to those in English although it is unlikely that other non-English peer review articles exist as reference lists of all articles were also screened. There is a possibility that research examining parkrun not published in English exist, so our conclusion that the research base is limited to a few countries, itself is limited. It is clear that this scoping review summarises research in its infancy; the first published paper on parkrun was ten years after its inception and the majority (n = 11) were published in 2018/2019.

## 5 Conclusion and future research

The current literature on parkrun suggests there are preliminary indications of public health success in terms of reach and impact on the health and wellbeing of participants. It does appear that as an organically grown initiative it has taken time for it to become “visible” enough to capture the attention of the scientific community - the existing (English language) evidence is currently limited to the UK and Australian contexts. The research also focusses on those who have benefitted from parkrun and therefore our knowledge is only partial; we know why it works for those it works for. As yet there is no published data on why others do not participate or benefit in the same way. Although the parkrun Research Board’s listing of currently live projects (Advanced Wellbeing Research Centre, 2020a) shows potential for wider coverage of important subpopulations (walkers, older women, people with a disability or chronic disease, volunteers) and topics (evaluations of linkages to primary care and junior parkrun) in future, many under-researched aspects still exist and addressing them would not only advance knowledge in the area of physical activity intervention but also inform parkrun operations. Below we outline key areas and gaps for future research on parkrun highlighted by our review, namely under-researched groups, more definitive long-term assessment of impact and system level analyses.

The largest groups absent from the peer reviewed research to date are: 1) People who do not register for parkrun; 2) people who register for parkrun and do not participate; 3) people who participate in parkrun but do not sustain it. Research in these populations would be key to parkrun achieving its aim of inclusivity. Future studies could use a sample of the general community and/or non-participants to assess awareness of parkrun, initial appeal of the format and ethos, and any perceived or real impediments to participation. Comparative studies of examining those who discontinue attending parkrun could be informative about failure in retention. As a key feature of population level intervention sustainability (Reis et al., 2016), future research should continue to monitor inclusivity and investigate how various retention strategies contribute to achieving parkrun and public health objectives. Assessing the effectiveness of various retention strategies, such as PROVE, could include comparator groups to better attribute causality (Quirk and Haake, 2019). Expanding the research beyond English-speaking countries would also improve our understanding of cultural relevance and specificity of the parkrun model.

Measuring the true extent of parkrun’s impact includes exploring the neurobiological, psychosocial and behavioural mechanisms that purportedly link physical activity and mental wellbeing outcomes (Lubans et al., 2016). However, quantifying the impact of parkrun is somewhat hindered by the ability to identify suitable comparison groups. Building on the existing process evaluations of parkrun implementation strategies, employing longitudinal study designs with appropriate comparators will triangulate the quasi-experimental studies most often used to assess the effectiveness of parkrun. Further investigation is needed of its contribution to total physical activity levels and the potential to stimulate wider participation in physical activity and sport given the high prior activity estimates of participants. Continued long term follow-up of participants would confirm the benefit to individuals’ health, particularly if linked to administrative datasets documenting health service utilisation and morbidity. Economic evaluation would quantify the cost benefit of parkrun, a key component of public health investment decision making (Ding et al., 2019).

Whilst there has been good attention to individual level mechanisms for participation maintenance (Wiltshire and Stevinson, 2018, Stevinson et al., 2015, Wiltshire et al., 2018, Cleland et al., 2019), evaluating program maintenance at the system level is also fundamental to assessing the public health value of parkrun (Reis et al., 2016). Rigorous monitoring and evaluation of how different models of dissemination operate in various cultural contexts would inform intervention adoption and adaption in the real world. It is evident that the components of parkrun do not work in isolation and although the organisers characterise parkrun as a social intervention it is not unimportant that it is grounded in physical activity. As a multi-component intervention with a range of interacting parts and with multiple local adaptations, future research could examine the emergent properties of parkrun (Craig et al., 2008) at the broader economic and cultural impact level. Finally, comparing parkrun with other interventions will yield insights into program translation and the critical combination of parkrun components unique to its success.

## Declaration of Competing Interest

Dr. Reece reports grants from parkrun Australia, outside the submitted work and is a member of the Global parkrun Research Board and co-ordinates research requests for parkrun Australia for which she receives no financial payment. Dr Grunseit and Dr Reece are registered parkrunners.
